# COVID-related delays in non-urgent adult surgeries: comparing population-based results from two Canadian provinces

**DOI:** 10.3389/fsurg.2025.1591265

**Published:** 2025-07-28

**Authors:** Rui Fu, Qing Li, Andrew Calzavara, Khara Sauro, Antoine Eskander

**Affiliations:** ^1^ Departments of Community Health Sciences, Surgery & Oncology, Cumming School of Medicine, University of Calgary, Calgary, AB, Canada; ^2^ ICES, Toronto, ON, Canada; ^3^ Department of Otolaryngology – Head and Neck Surgery, University of Toronto, Toronto, ON, Canada; ^4^ Institute of Health Policy, Management and Evaluation, Dalla Lana School of Public Health, University of Toronto, Toronto, ON, Canada

**Keywords:** surgery, COVID-19, pandemic, public health policy, outcomes

## Abstract

**Background:**

During the COVID-19 pandemic, non-urgent surgeries were delayed in order to increase the capacity to care for patients with COVID-19. To shed light on the effect of pandemic-related surgical ramp down on the quality of surgical care, this study compared Ontario with Alberta on (1) changes in the proportion of completion and wait time of surgeries with decision-to-treat in a pre-pandemic period compared to those with decision-to-treat in each of the four COVID-19 waves and (2) shifts in healthcare utilization and safety of surgical patients for the same time periods.

**Methods:**

A retrospective population-based cohort study was conducted in Ontario on scheduled non-urgent surgeries among adults with decision-to-treat (index dates) between January 1, 2018 and December 31, 2021. Logistic regression was used to examine surgery completion (observed up to December 31, 2021) on the index date period (each COVID-19 wave vs. pre-pandemic). For completed surgeries, median regression was used to assess wait time on the index date period. Descriptive statistics were provided on healthcare utilization and safety indicators among the cohort. Results from regression models and descriptive statistics were then compared with published data from Alberta.

**Results:**

There were 2,073,688 non-urgent surgeries scheduled for 1,560,265 unique adults in Ontario. Surgeries with an index date in each COVID-19 wave were associated with lower odds of completion compared to the pre-pandemic period, which is in contrast to Alberta where the odds of having surgery completed was not lower during the pandemic than pre-pandemic. Among completed surgeries (91.7%) in Ontario, the median wait time was shorter for surgeries with an index date in waves 2 and 4 than in the pre-pandemic period, while in Alberta the median wait time was shorter for surgeries with index dates in waves 2–4 than pre-pandemic. During the pandemic, Alberta reported a decrease in median intensive care unit (ICU) hours and hospital length of stay for patients relative to pre-pandemic, while Ontario reported an increase in median ICU hours of these patients.

**Conclusions:**

These findings highlight interprovincial differences in surgical care which might be related to COVID-19 policies in each province, healthcare system capacity and patient demographics.

## 1 Introduction

On January 25, 2020, Canada's first case of COVID-19 was confirmed in Ontario. On March 15, 2020, Ontario hospitals were directed to halt non-emergent procedures (1). Most provinces had similar directives; for example, Alberta announced a suspension of non-urgent surgeries on March 18, 2020 (2). Following these directives, strategic resumption of surgical care started in early May 2020 for both Ontario and Alberta with varying policies. A recent environmental review (3) highlighted unique strategies implemented by Alberta to maintain surgical care delivery amid COVID-19 which included expanding the network of accredited private institutions; opening new operating rooms in public hospitals; and conversion to a centralized intake for certain surgery types (e.g., orthopedic and general) (2). With the implementation of these novel strategies, reports suggest Alberta reached 88% of its pre-pandemic surgical volume 5 months into the pandemic (2), and as of August 2021, 100% of the backlogged surgeries had been rebooked and 96% completed (4). At the same time (August 2021), surgical backlog incurred in Ontario was estimated to take at least another 22 weeks to clear (5). These preliminary results suggest Alberta may have been able to maintain surgical capacity better than Ontario during early pandemic and also achieved a faster recovery, but this has not been formally examined in an inter-provincial comparative analysis using reliable population-based data sources from both provinces.

Following the same methodology used in a recent Alberta study (6), we examined Ontario adults with decision-to-treat between January 1, 2018 and December 31, 2021 for a non-urgent surgery to: (1) assess surgery completion and wait times during four distinct COVID-19 waves compared to a pre-pandemic period (based on the decision-to-treat date); (2) describe healthcare utilization and safety indicators of these surgical patients, and (3) compare the findings from Ontario with Alberta.

## 2 Materials and methods

### 2.1 Study design and data sources

This two-part study included: (1) a retrospective population-based cohort study using linked health administrative data in Ontario and (2) a systematic comparison of findings from the Ontario cohort study with those from a comparable cohort study conducted in Alberta (6).

For the Ontario cohort study, seven health administrative datasets were linked using unique encoded identifiers and analyzed at ICES (previously known as the Institute for Clinical Evaluative Sciences). Ontario residents have universally accessible and publicly funded health care through the Ontario Health Insurance Plan (OHIP). The study cohort was identified using the Wait Time Information System (WTIS), which is a validated web-based application that tracks the wait time for surgery and diagnostic imaging in Ontario hospitals (7). At the time of analysis (January-July 2024), the surgery subset of WTIS (i.e., the WTIS surgery subset) contained reliably up-to-date data on decision-to-treat date, surgical procedure, and procedure date until October 2023. The WTIS surgery subset was deterministically linked to six datasets: (1) The Registered Persons Database contains demographic information on residents of Ontario covered under OHIP. (2) The Office of the Registrar General provided vital statistics. (3) The OHIP claims database contains information on physician billings, including patient and physician identifiers (encrypted), code for service provided, date of service, associated diagnosis, and fee paid. (4) The Discharge Abstract Database (DAD) includes records of all acute care hospital admissions including intensive care unit (ICU) admissions. (5) The Same-Day Surgery (SDS) database has records of day surgeries performed at hospital. (6) The National Ambulatory Care Reporting System identifies hospital- and community-based ambulatory care procedures, including emergency department (ED) visits. Further information on the datasets is included in Supplementary Table S1.

### 2.2 Defining surgical procedures and constructing the cohort

A surgical procedure was defined as a unique combination of a patient identifier (OHIP number), a decision-to-treat date, a service area (high-level category of the surgical field/specialty), and a procedure date (if the surgery had taken place by December 31, 2021) from the WTIS surgery subset. Date of decision-to-treat (index date) was the date when a patient consented to the procedure deemed necessary by the surgeon and was placed on the waiting list. The study cohort included all non-urgent surgical procedures scheduled for an OHIP-eligible adult with an index date between January 1, 2018 and December 31, 2021. If a patient had multiple eligible procedures, we included all of them to allow for a procedure-level (rather than a patient-level) analysis, to stay consistent with the Alberta study (6). Procedures involving a patient with age<18 or >105 years, with an invalid patient identifier, an “emergent” priority status (8, 9), or an ED visit on the procedure date (interpreted to be an urgent surgery) were excluded (see flow diagram in Supplementary Figure S1). Each procedure was followed from the index date to procedure date, date of death, or December 31, 2021; whichever occurred first.

### 2.3 Primary exposure – index date period

For the Ontario analysis, index dates were classified into the following periods using the Public Health Ontario definition of COVID-19 pandemic waves (10): pre-pandemic (January 1, 2018–February 29, 2020), wave 1 (March 1, 2020–August 29, 2020), wave 2 (August 30, 2020–February 27, 2021), wave 3 (February 28, 2021–July 31, 2021), and wave 4 (August 1, 2021–December 31, 2021).

### 2.4 Outcomes

The primary outcome was surgery completion, which was a binary variable denoting if a surgery was complete as of December 31, 2021, using the WTIS surgery subset (6). Four sets of secondary outcomes were accessed: (1) for complete surgeries, surgical wait time was calculated as the number of days from the index date to the procedure date. (2) Rates (number of occurrences per patient-year) of hospital admissions, 30-day readmissions, ED visits, and physician visits were calculated by counting healthcare utilization for each surgery from the index date to procedure date, death date, or December 31, 2021; whichever occurred first (numerator), and divided it by the total number of patient-years included in the pre-pandemic period and in the COVID-19 pandemic (denominator). If a patient had multiple hospital admissions or ED/physician visits during a time period, we included all occurrences in the numerator and counted this patient once in the denominator. (3) Mean and median hospital length of stay (number of days), ICU hours, and Resource Intensity Weights associated with each surgical hospital admission (11) were reported for each time period. (4) For safety indicators, rates (number of occurrences per patient-year) of at least one in-hospital complication (12), in-hospital death, and all-cause death were reported for each time period.

### 2.5 Covariates

Patient age on the index date, sex, surgery type, region of institution, and surgery priority level were extracted from the WTIS surgery subset. Surgery priority level was reported by surgeons using a standard protocol to classify each surgery on the date of decision-to-treat into the following priority groups: Priority I/Emergent (target of 24 h from decision to surgery; not included in this study), Priority II (target of 1–8 weeks from decision), Priority III (4–16 weeks from decision), and Priority IV (12–26 weeks from decision) (8, 9). Charlson Comorbidity Index was calculated using a 5-year look back window at the index date, and categorized as 0, 1, 2 and above; a separate category was created for those who had no DAD or SDS records in the past 5-years.

### 2.6 Statistical analysis

Characteristics of surgeries with decision-to-treat (index dates) during the COVID-19 pandemic (combining waves 1–4; March 1, 2020–December 31, 2021) vs. pre-pandemic (January 1, 2018–February 29, 2020) were compared using a standardized mean difference (SMD) of >0.10 to indicate a notable imbalance in variable distribution between the two groups (13). A multivariable logistic regression model was used to assess the association between the primary outcome (binary variable of surgery completion) and each index date period (6). The regression analysis was then repeated for each surgery type. For completed surgeries, a median regression was used to assess the association between wait time and the index date period; coefficient estimates are interpreted as the change in the median wait time (number of days) for surgeries scheduled in a COVID-19 wave compared to the pre-pandemic period. For healthcare utilization and safety indicators, rates (number of occurrences per patient-year), means with standard deviation (±SD), or medians with interquartile range (IQR) were summarized for the pre-pandemic period and during the pandemic (combining waves 1–4) based on the timing of utilization or adverse events (for safety indicators). Plots were created to visually and systematically compare the Ontario results with those of Alberta (6). Analyses were two-sided, and statistical significance was set at *p*-value<0.05. Analyses were performed on SAS 9.4 (SAS Institute). Visualizations were created on Excel version 16.89.1 (Microsoft 365).

## 3 Results

### 3.1 Ontario results

There were 2,073,688 non-urgent surgeries scheduled for 1,560,265 unique adults over 2018–2021 in Ontario. Of them, 40.0% (*n* = 830,479) had an index date during the COVID-19 pandemic. There were 7.4% (*n* = 154,145) surgeries with an index date during wave 1, 12.4% (*n* = 257,798) in wave 2, 10.0% (*n* = 208,064) in wave 3, and 10.1% (*n* = 210,472) in wave 4. When comparing surgeries with an index date during the COVID-19 pandemic to those scheduled during pre-pandemic period (Table 1), their characteristics were largely similar, except more surgeries were classified as Priority II (6.6% vs. 3.4%, SMD = 0.15) or Priority III (28.9% vs. 22.2%, SMD = 0.15) during the COVID-19 pandemic compared to the pre-pandemic period with fewer Priority IV surgeries during the pandemic compared to the pre-pandemic period (64.5% vs. 74.4%, SMD = 0.22).

**Table 1:** Characteristics of non-urgent surgeries scheduled for an Ontario adult before and after the start of the COVID-19 pandemic.

Variable	Pre-pandemic *n* = 1,243,209, 60.0%	Pandemic (waves 1–4) *n* = 830,479, 40.0%	Total *n* = 2,073,688	Standardized mean difference
Age at decision-to-treat				
Mean ± SD, year	59.12 ± 16.77	59.82 ± 16.72	59.40 ± 16.75	0.04
Age group				
18–49	341,838 (27.5%)	215,971 (26.0%)	557,809 (26.9%)	0.03
50–64	362,002 (29.1%)	234,563 (28.2%)	596,565 (28.8%)	0.02
65–74	305,869 (24.6%)	216,242 (26.0%)	522,111 (25.2%)	0.03
75+	233,500 (18.8%)	163,703 (19.7%)	397,203 (19.2%)	0.02
Sex				
Female	697,348 (56.1%)	460,572 (55.5%)	1,157,920 (55.8%)	0.01
Male	545,861 (43.9%)	369,907 (44.5%)	915,768 (44.2%)	0.01
Charlson comorbidity group^a^				
0	547,712 (44.1%)	358,443 (43.2%)	906,155 (43.7%)	0.02
1	99,956 (8.0%)	65,001 (7.8%)	164,957 (8.0%)	0.01
2+	188,319 (15.1%)	133,485 (16.1%)	321,804 (15.5%)	0.03
No DAD/SDS in past 5 years	407,222 (32.8%)	273,550 (32.9%)	680,772 (32.8%)	0
Type of surgery				
General	181,851 (14.6%)	120,237 (14.5%)	302,088 (14.6%)	0
Gynaecologic	128,754 (10.4%)	79,254 (9.5%)	208,008 (10.0%)	0.03
Neurosurgery	16,653 (1.3%)	10,671 (1.3%)	27,324 (1.3%)	0
Oncology	117,148 (9.4%)	91,811 (11.1%)	208,959 (10.1%)	0.05
Ophthalmic	273,876 (22.0%)	190,385 (22.9%)	464,261 (22.4%)	0.02
Oral, maxillofacial, dentistry	22,035 (1.8%)	10,535 (1.3%)	32,570 (1.6%)	0.04
Orthopaedic	258,040 (20.8%)	168,706 (20.3%)	426,746 (20.6%)	0.01
Otolaryngology	61,439 (4.9%)	38,042 (4.6%)	99,481 (4.8%)	0.02
Plastic and Reconstructive	57,660 (4.6%)	32,963 (4.0%)	90,623 (4.4%)	0.03
Thoracic	3,958 (0.3%)	2,772 (0.3%)	6,730 (0.3%)	0
Urologic	97,004 (7.8%)	67,298 (8.1%)	164,302 (7.9%)	0.01
Vascular	24,791 (2.0%)	17,805 (2.1%)	42,596 (2.1%)	0.01
Region of the institution^b^				
West	387,825 (31.2%)	266,200 (32.1%)	654,025 (31.5%)	0.02
Central	237,064 (19.1%)	152,384 (18.3%)	389,448 (18.8%)	0.02
Toronto	289,367 (23.3%)	186,938 (22.5%)	476,305 (23.0%)	0.02
East	245,484 (19.7%)	169,149 (20.4%)	414,633 (20.0%)	0.02
Northeast	59,178 (4.8%)	38,191 (4.6%)	97,369 (4.7%)	0.01
Northwest	23,632 (1.9%)	16,680 (2.0%)	40,312 (1.9%)	0.01
Priority level^c^				
II (target completion in 1–8 weeks)	42,199 (3.4%)	54,782 (6.6%)	96,981 (4.7%)	0.15
III (target completion in 4–16 weeks)	276,080 (22.2%)	239,873 (28.9%)	515,953 (24.9%)	0.15
IV (target completion in 12–26 weeks)	924,930 (74.4%)	535,824 (64.5%)	1,460,754 (70.4%)	0.22

We measured the characteristics of each surgery on decision-to-treat (index date) using the following index date periods: pre-pandemic (January 1, 2018–February 29, 2020) and the pandemic (March 1, 2020–December 31, 2021). A standardized mean difference > 0.10 was used to indicate a notable imbalance in variable distribution of the two groups.

SD, standard deviation; DAD, Discharge Abstract Database; SDS, Same-Day Surgery; SMD, standardized mean difference.

^a^ Comorbidity was computed using records from the Hospital Discharge Abstract Database and Same-Day Surgery database over a 5-year period before the index date. Those with no records were grouped separately from patients scored 0, 1, or 2+ on the Charlson Comorbidity Index.

^b^ Around 0.1% of records had missing data for region of the institution; the distribution did not differ significantly between the two groups.

^c^ A standard protocol exists for Ontario surgeons to determine the priority level of each surgery at the decision-to-treat. Surgeries deemed to have Priority I/Emergent (target of 24 h from decision to surgery) were excluded from the present analysis.

As of December 31, 2021, 85.7% (*n* = 1,777,877) of the scheduled surgeries in Ontario were completed, including 91.9% (*n* = 1,141,977) of surgeries with an index date during pre-pandemic period and 76.6% (*n* = 635,900) of surgeries with an index date during the COVID-19 pandemic (Supplementary Table S2). Using logistic regression, it was found that the odds of having a surgery completed was lower for surgeries with an index date during all COVID-19 waves compared to surgeries with an index date during the pre-pandemic period; the adjusted odds ratio of having a surgery completed during wave 1 (compared to the pre-pandemic period) was 0.682 (95% CI 0.670–0.694), during wave 2 was 0.641 (95% CI 0.632–0.650), during wave 3 was 0.312 (95% CI 0.308–0.316), and during wave 4 was 0.078 (95% CI 0.077–0.079) (Supplementary Table S3). When repeating the analysis for each surgery type, it was found that having an index date during the COVID-19 pandemic was associated with a lower odds of having surgery completed than having an index date during the pre-pandemic period for all types of surgery (Supplementary Table S4).

Of all completed surgeries as of December 31, 2021 in Ontario, the median wait time (from index date to surgery date) was 52 (IQR, 25–110) days for those with an index date in pre-pandemic period and 41 (IQR, 20–96) days for those with an index date in the pandemic period (Supplementary Table S5). Using multivariable median regression, surgeries having an index date in wave 1 (8.8, 95% CI 8.4–9.2) and wave 3 (5.3, 95% CI 5.0–5.6) had a higher median wait time (number of days) than those with an index date during the pre-pandemic period, while surgeries with an index date during wave 2 (−6.6, 95% CI −6.8 to −6.4) or wave 4 (−16.0, 95% CI −16.2 to −15.7) had a lower median wait time than those with an index date in the pre-pandemic period (Supplementary Table S6).

Among the Ontario cohort (Table 2), the rate of hospital admissions decreased from 0.99 per patient-year in the pre-pandemic period to 0.57 per patient-year during the COVID-19 pandemic. Similar patterns were observed for the rate of 30-day readmissions, ED visits, and physician visits. Conversely, hospital length of stay, ICU hours and resource intensity were greater during the COVID-19 pandemic compared to the pre-pandemic period. For patient safety indicators, while the rate of having a hospital admission, an in-hospital complication, or in-hospital mortality decreased from the pre-pandemic period to the COVID-19 pandemic, the rate of all-cause mortality increased from 0.06 per patient-year in the pre-pandemic period to 0.09 per patient-year during the pandemic.

**Table 2:** Healthcare utilization and safety for Ontario patients with a surgery scheduled during 2018–2021.

Variable	Pre-pandemic	Pandemic (waves 1–4)	Total
Number of patient-year	330,901.5	452,038.2	782,939.7
Healthcare utilization			
Number of visits (per patient-year)			
Hospital admissions	327,739 (0.99)	258,142 (0.57)	585,881 (0.75)
30-day hospital readmissions	20,439 (0.06)	21,728 (0.05)	42,167 (0.05)
Emergency department visits	253,038 (0.76)	306,071 (0.68)	559,109 (0.71)
Physician visits			
Total visits	11,552,439 (34.91)	11,988,631 (26.52)	23,541,070 (30.07)
GP – Primary care visits	1,727,369 (5.22)	2,202,655 (4.87)	3,930,024 (5.02)
GP – Non-primary care visits	1,627,701 (4.92)	1,894,874 (4.19)	3,522,575 (4.50)
Specialist visits	8,197,369 (24.77)	7,891,102 (17.46)	16,088,471 (20.55)
Utilization at admission level			
Hospital length of stay, day, mean ± SD	3.75 ± 9.19	4.24 ± 9.46	3.97 ± 9.31
ICU hours, hour, mean ± SD	75.33 ± 161.39	85.51 ± 189.74	79.85 ± 174.62
Resource intensity, mean ± SD	1.40 ± 2.01	1.46 ± 2.16	1.42 ± 2.08
Patient safety indicators			
Number of patients (per patient-year)			
Had a hospital admission	278,217 (0.84)	213,693 (0.47)	461,719 (0.59)
Had an at-hospital complication	21,788 (0.07)	17,985 (0.04)	39,231 (0.05)
Died at hospital	2,879 (0.009)	3,775 (0.008)	6,654 (0.008)
All-cause mortality	20,119 (0.06)	41,641 (0.09)	61,760 (0.08)

We followed each surgery from the date of decision-to-treat to the date of surgery, date of death, or end of wave 4 (December 31, 2021); whichever occurred first, to capture healthcare utilization and safety outcomes. If a patient had multiple surgeries scheduled between January 1, 2018 and December 31, 2021, we counted the healthcare utilization and adverse events (for safety outcomes) associated with each surgery. If a patient had multiple hospital admissions or emergency department/physician visits associated with a surgery during a time period, we counted all occurrences in the numerator and counted this patient once in the denominator (for calculating rates). Time periods (columns) are based on the timing of utilization or an adverse event (for safety indicators), not the index (decision-to-treat) date. To calculate rates, the denominator represents the total patient-year in each time period. GP, general practitioner; SD, standard deviation; ICU, intensive care unit.

### 3.2 Comparison with Alberta

Between January 2018 and December 2021, there were 259,677 non-urgent surgeries scheduled for 202,470 unique adults in Alberta. This represents 12.5% and 13.0% of the levels of Ontario, respectively. Of these surgeries, 12.1% (vs. 10.1% in Ontario, *p*-value<0.01) were cancer surgeries. Patients were younger in Alberta (56.2 ± 17.0 years at index date) than in Ontario (59.4 ± 16.8 years, *p*-value<0.01).

**Figure 1:**
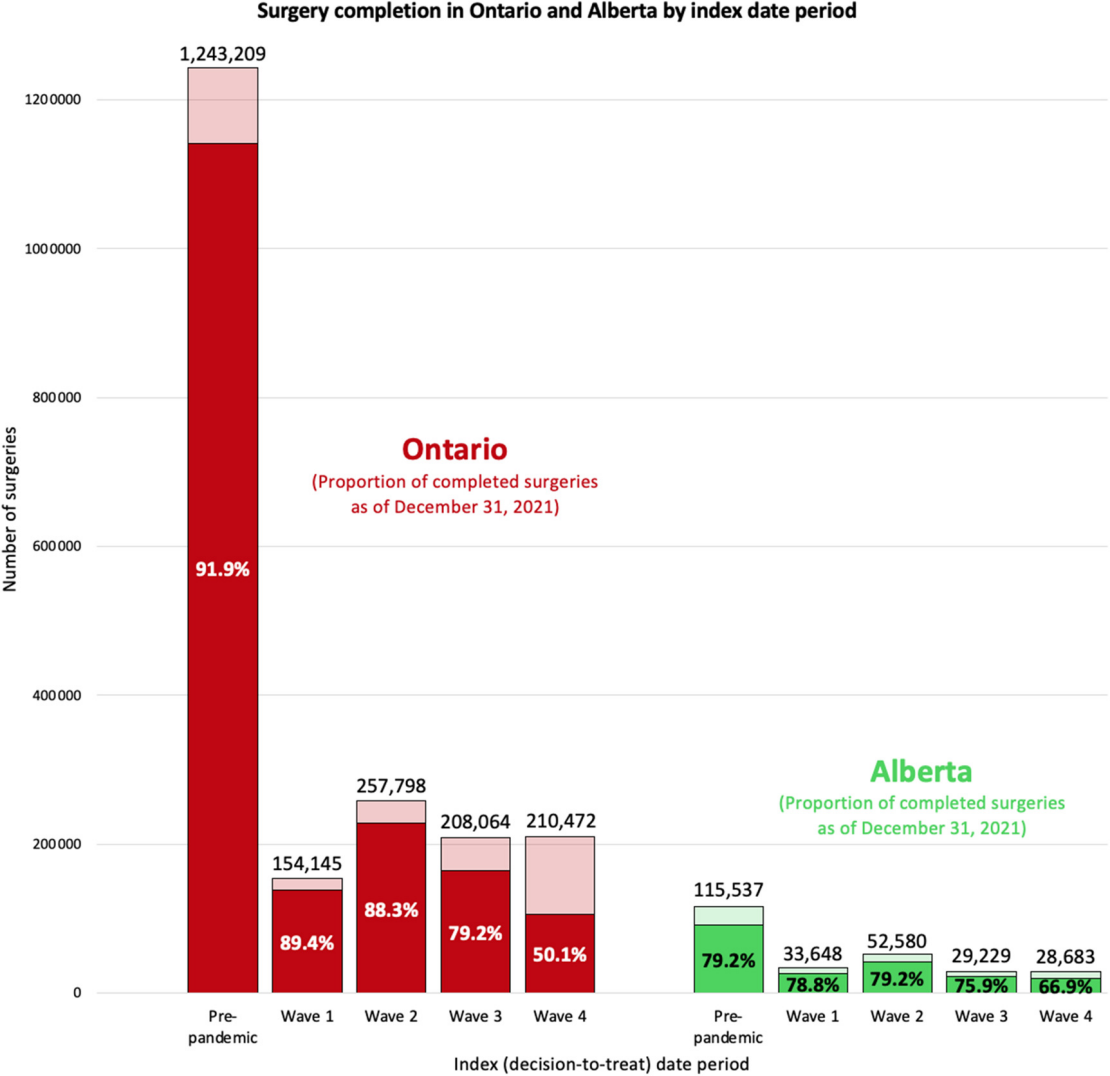
Compare surgery ncompletion in Ontario and Alberta by index date period. We report the number of surgeries with decision-to-treat in each index date period and the proportion of completed surgeries as of December 31, 2021. Index (decision-to-treat) dates are categorized into the following periods: Alberta: pre-pandemic (January 1, 2018–February 29, 2020), wave 1 (March 1, 2020–August 22, 2020), wave 2 (August 23, 2020–March 20, 2021), wave 3 (March 21, 2021–July 17, 2021), and wave 4 (July 18, 2021–December 31, 2021); Ontario: pre-pandemic (January 1, 2018–February 29, 2020), wave 1 (March 1, 2020–August 29, 2020), wave 2 (August 30, 2020–February 27, 2021), wave 3 (February 28, 2021–July 31, 2021), and wave 4 (August 1, 2021–December 31, 2021).

**Figure 2:**
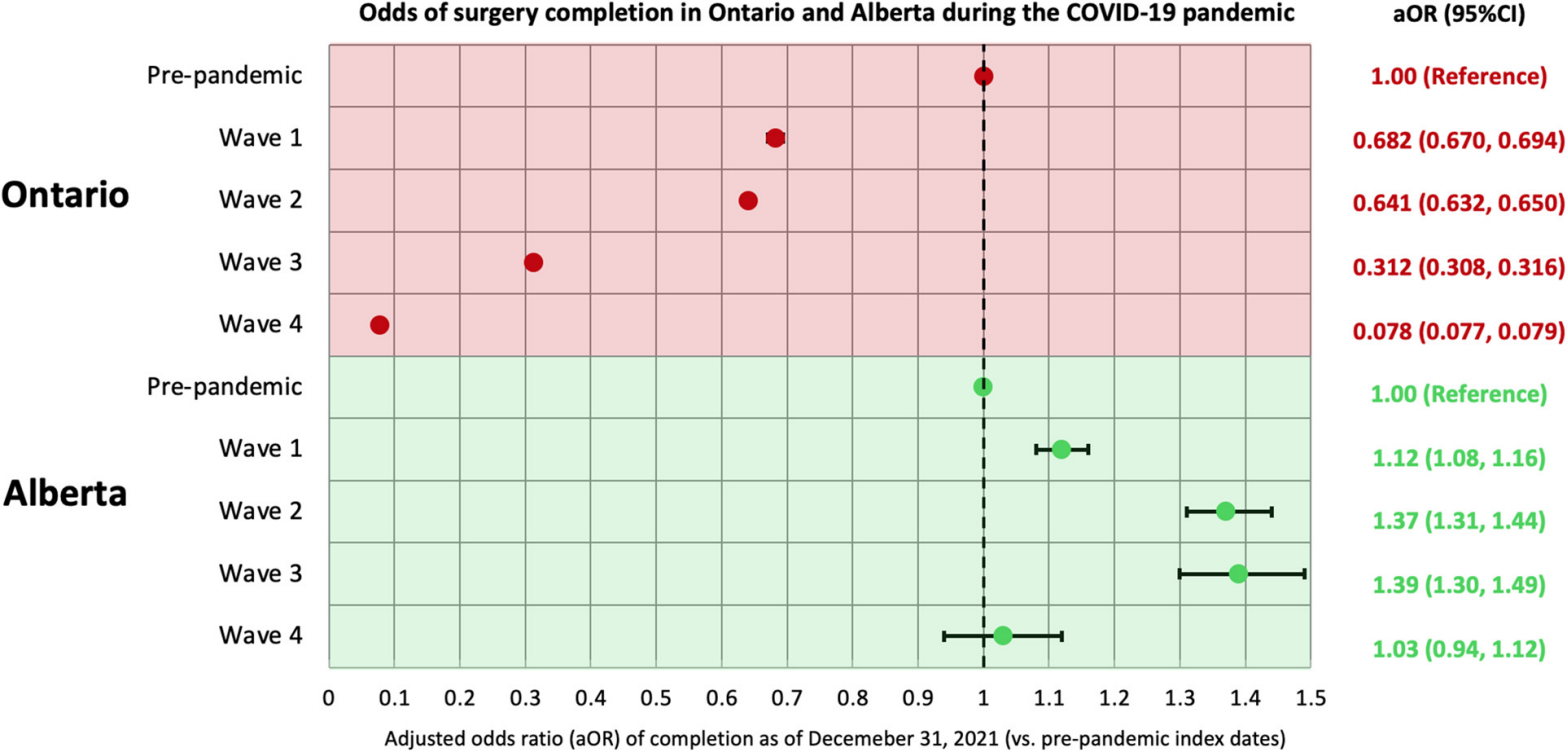
Odds of non-urgent adult surgery completion in Ontario and Alberta during the COVID-19 pandemic. Index (decision-to-treat) dates are categorized into the following periods: Alberta: pre-pandemic (January 1, 2018–February 29, 2020), wave 1 (March 1, 2020–August 22, 2020), wave 2 (August 23, 2020–March 20, 2021), wave 3 (March 21, 2021–July 17, 2021), and wave 4 (July 18, 2021–December 31, 2021); Ontario: pre-pandemic (January 1, 2018–February 29, 2020), wave 1 (March 1, 2020–August 29, 2020), wave 2 (August 30, 2020–February 27, 2021), wave 3 (February 28, 2021–July 31, 2021), and wave 4 (August 1, 2021–December 31, 2021). In both analyses, a multivariable logistic regression model was used to assess the odds of surgery being completed up to December 31, 2021 in relation to the index date period, accounting for patient age, sex, comorbidity, surgery type, region of surgical care institution, and surgery priority level. aOR, adjusted odds ratio; CI, confidence interval.

As of December 31, 2021, 79.2% (*n* = 91,473/115,537) of surgeries with an index date in the pre-pandemic period and 76.0% (*n* = 109,588/144,140) of surgeries with an index date during the COVID-19 pandemic were completed in Alberta, both of which were lower than proportion of surgeries completed in Ontario (91.9% and 76.6%; Figure 1; Supplementary Table S2). Using results from a similar multivariable logistic regression model, the odds of having a surgery completed in Alberta was significantly greater for those with an index date in the first three waves of the COVID-19 pandemic than those with an index date during the pre-pandemic period; there was no difference in the odds of having surgery completed in Alberta for those with an index date during wave 4 compared to those with an index date during the pre-pandemic period. This is in contrast with the findings from Ontario where the odds of having a surgery completed during the COVID-19 pandemic (all waves) was lower than those with an index date during the pre-pandemic period (Figure 2).

**Figure 3:**
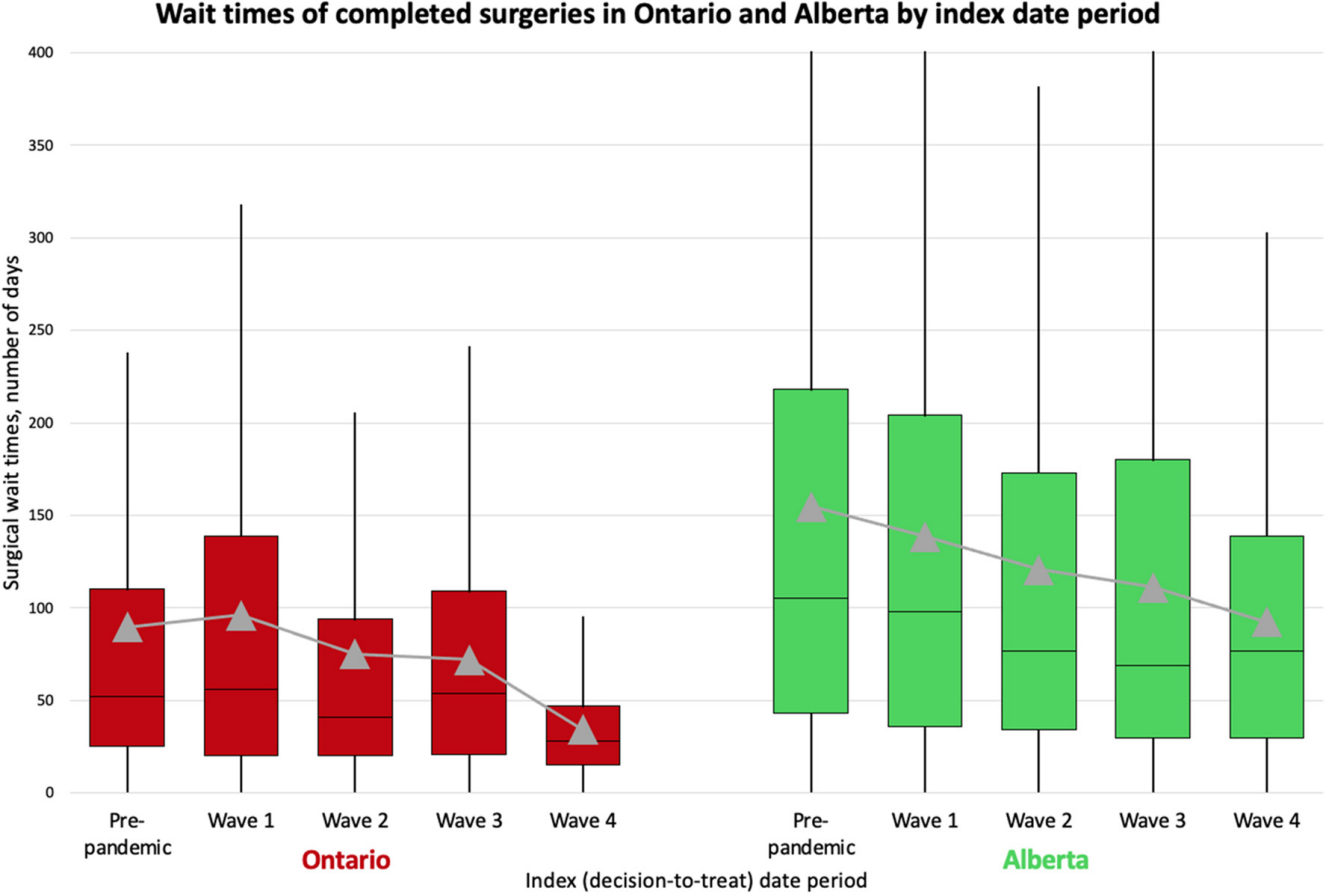
A box-and-whisker plot showing wait times of completed surgeries in Ontario and Alberta by index date period. We present the distribution of wait times by index date period including mean wait times (grey triangles). Index (decision-to-treat) dates are categorized into the following periods: Alberta: pre-pandemic (January 1, 2018–February 29, 2020), wave 1 (March 1, 2020–August 22, 2020), wave 2 (August 23, 2020–March 20, 2021), wave 3 (March 21, 2021–July 17, 2021), and wave 4 (July 18, 2021–December 31, 2021); Ontario: pre-pandemic (January 1, 2018–February 29, 2020), wave 1 (March 1, 2020–August 29, 2020), wave 2 (August 30, 2020–February 27, 2021), wave 3 (February 28, 2021–July 31, 2021), and wave 4 (August 1, 2021–December 31, 2021).

**Figure 4:**
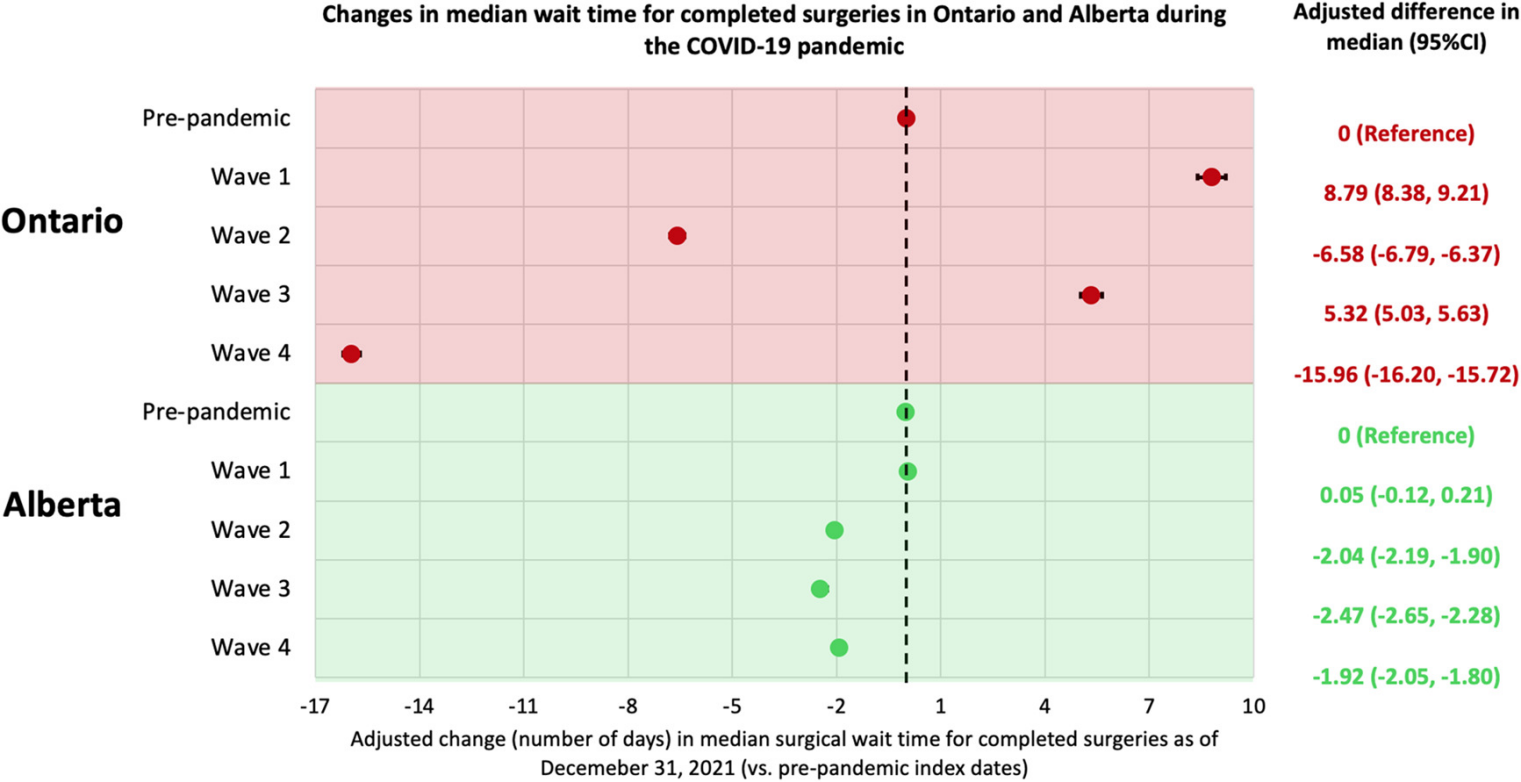
Changes in median wait times for completed surgeries in Ontario and Alberta during the COVID-19 pandemic. Index (decision-to-treat) dates are categorized into the following periods: Alberta: pre-pandemic (January 1, 2018–February 29, 2020), wave 1 (March 1, 2020–August 22, 2020), wave 2 (August 23, 2020–March 20, 2021), wave 3 (March 21, 2021–July 17, 2021), and wave 4 (July 18, 2021–December 31, 2021); Ontario: pre-pandemic (January 1, 2018–February 29, 2020), wave 1 (March 1, 2020–August 29, 2020), wave 2 (August 30, 2020–February 27, 2021), wave 3 (February 28, 2021–July 31, 2021), and wave 4 (August 1, 2021–December 31, 2021). In both analyses, a multivariable median regression model was used to assess the relationship between wait times (number of days) and the index date period, accounting for patient age, sex, comorbidity, surgery type, region of surgical care institution, and surgery priority level. CI, confidence interval.

**Figure 5:**
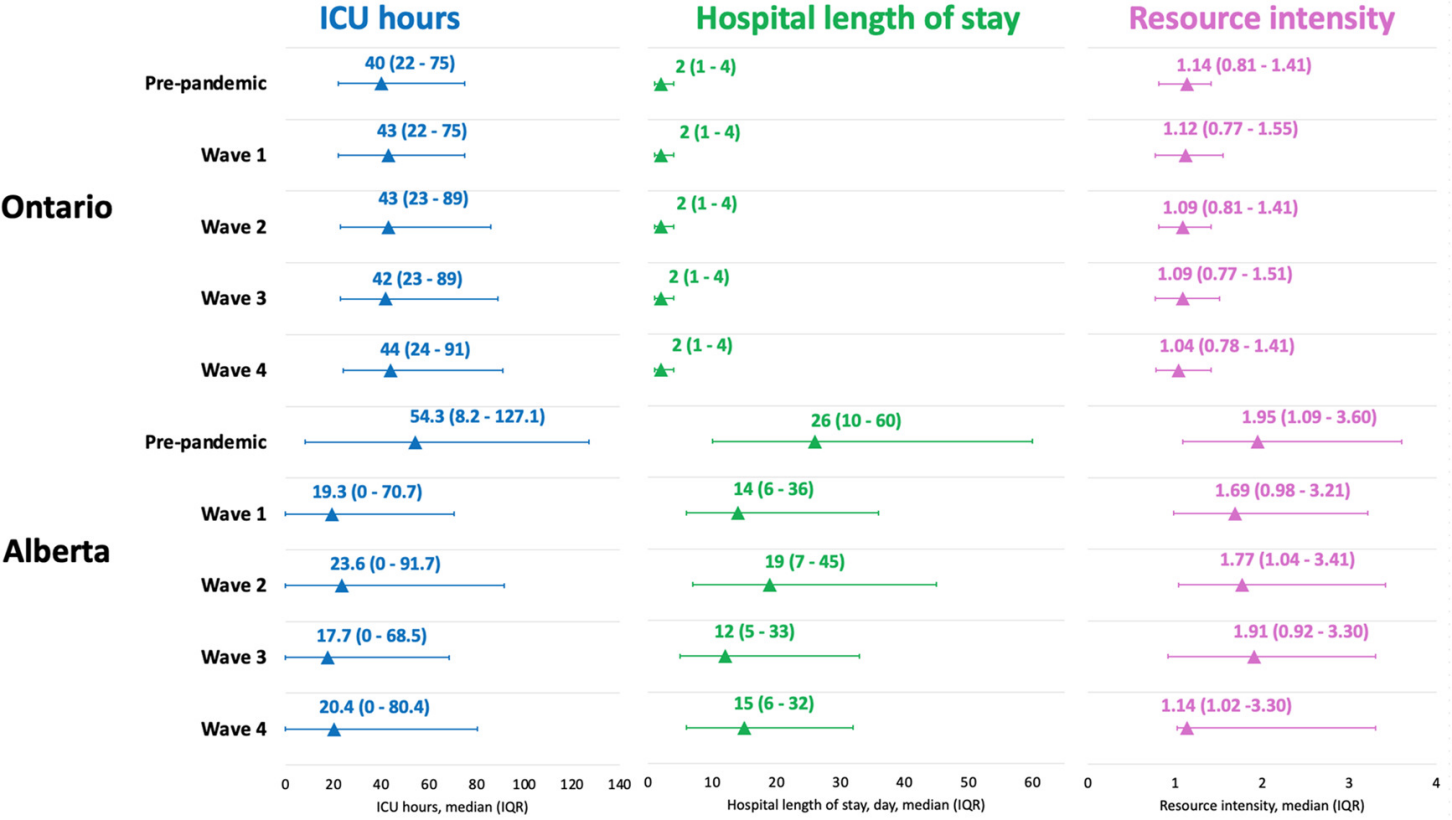
Compare healthcare utilization among patients with a surgery scheduled between Jan2018-Dec2021 in Ontario and Alberta. Timing of healthcare utilization are categorized into the following periods: Alberta: pre-pandemic (January 1, 2018–February 29, 2020), wave 1 (March 1, 2020–August 22, 2020), wave 2 (August 23, 2020–March 20, 2021), wave 3 (March 21, 2021–July 17, 2021), and wave 4 (July 18, 2021–December 31, 2021); Ontario: pre-pandemic (January 1, 2018–February 29, 2020), wave 1 (March 1, 2020–August 29, 2020), wave 2 (August 30, 2020–February 27, 2021), wave 3 (February 28, 2021–July 31, 2021), and wave 4 (August 1, 2021–December 31, 2021). We reported the median and interquartile range for each type of utilization. ICU, intensive care unit; IQR, interquartile range.

Among completed surgeries in Alberta, the median wait time was 105 (IQR 43–218) days for those with an index date in the pre-pandemic period and 80 (IQR 33–173) days for those with an index date during the COVID-19 pandemic, which is double the wait time in Ontario for both periods (52 days and 41 days, Figure 3; Supplementary Table S5). Using the results from similar median regression models, the median wait time in Alberta was around 2-days shorter for surgeries with an index date in waves 2, 3 and 4 compared to that of surgeries with an index date in the pre-pandemic period. These findings are partially consistent with Ontario, where the median wait time was shorter for surgeries with an index date in wave 2 and wave 4 than surgeries with an index date in the pre-pandemic period (Figure 4). When looking at healthcare utilization and safety indicators of patients with at least one non-urgent surgery booked in 2018–2021, Alberta reported a lower median ICU length of stay (hours) and hospital length of stay (days) during the COVID-19 pandemic compared to the pre-pandemic period, while Ontario reported a longer median ICU length of stay (hours) during the COVID-19 pandemic compared to the pre-pandemic period and a stable median hospital length of stay (days) between the two time periods (Figure 5).

## 4 Discussion

Three main findings emerged from this population-based comparative study: (1) non-urgent surgeries scheduled in Ontario during all four waves of the COVID-19 pandemic (50.1%-89.4% completed) were less likely to be completed by December 2021 than those scheduled in the pre-pandemic period (91.9% completed), which differed from Alberta where the proportion of surgeries completed did not depend heavily on whether being scheduled during the pre-pandemic period (79.2% completed) or during COVID-19 pandemic (66.9%–79.2% completed). (2) Of completed surgeries, the median wait time was longer for surgeries scheduled in Ontario during wave 1 or wave 3 than those scheduled during the pre-pandemic period, while Alberta was able to lower its pre-pandemic wait times throughout the pandemic. Finally (3) among patients who were admitted to hospital for their surgery, Alberta reported a lower median ICU length of stay and hospital length of stay during the COVID-19 pandemic compared to the pre-pandemic period, while in Ontario there was a longer ICU length of stay during the COVID-19 pandemic compared to the pre-pandemic period. We hypothesize that these differences may be related to differences in COVID-19 containment and surgical policies, patient demographics and healthcare system capacity.

There was a noted increased capacity to complete surgeries in Alberta during the COVID-19 pandemic compared to Ontario. When examining the capacity to schedule surgeries (14), Alberta also showed an increase in the rate of scheduled surgeries from pre-pandemic to the pandemic period (from 122 to 184 per 10,000 persons) while Ontario reported a decrease in this rate (from 391 to 318 per 10,000 persons). These results may imply that Alberta had an enhanced capacity to schedule and complete surgeries during the COVID-19 pandemic compared to Ontario, which is likely multifactorial but may be related to a fortuitous investment by Alberta Health just 3 months before the COVID-19 pandemic. Alberta initiated the Surgical Wait Time Initiative, which included pilot testing the Facilitated Access to Specialized Treatment (FAST) program. The FAST program enabled a centralized referral intake for certain surgical types (i.e., orthopedic and general), which is an evidence-based mechanism with proven effectiveness in enhancing surgical booking and completion in Alberta (15, 16) and in other regions of North America, Europe and Australasia (17–19). Single centres in Ontario have experimented with centralized surgical intake but province-wide implementation had not occurred prior to, or during the COVID-19 pandemic, nor at the time of writing (9). The Alberta Surgical Wait Time Initiative also included contracting private surgical care centres to provide publicly funded surgeries towards the end of the COVID-19 pandemic which may have also contributed to the province's ability to clear the surgical waitlist (2). Ontario saw the official opening of its first non-for-profit free-standing surgical facility in March 2020; preliminary data suggests this facility delivered high-quality surgical care during the COVID-19 pandemic (20), which may have been the impetus for passing Bill 60 (Your Health Act) in 2023, which aims to expand the network of free-standing surgical centres, termed the Integrated Community Health Services Centres (21). A formal policy analysis is required to map the implementation of these novel strategies (centralized surgical referrals and allowing private providers to perform publicly funded surgery) with population outcomes to establish if these models of care may be viable options to making healthcare systems more resilient during a public health crisis.

Similarities and differences in wait times between provinces during the COVID-19 pandemic may also be related to policies and models of surgical care delivery in each province. Both provinces had shorter median surgical wait times for surgeries scheduled in wave 2 of the COVID-19 pandemic compared to those scheduled in the pre-pandemic period. This observation could be related to either a conversion of surgical procedures from non-urgent to urgent (i.e., patients who may not have needed an urgent surgery may have decompensated and required an urgent surgery for their illness) or may be attributed to physicians adopting non-surgical treatment and only reserving surgical care for the most urgent cases, as recommended in policies in both provinces (22, 23). Unlike Alberta, Ontario reported a 5-days longer median wait time in wave 3 compared to pre-pandemic. This may be attributed to differences in virus containment policies, as Ontario announced a third provincial lockdown and a second stay-at-home order in April 2021 (amid wave 3) that was in effect for one month, which was not the case in Alberta (24). It is worth noting that when comparing wait times between the two provinces (Supplementary Table S5; Figure 3), Alberta reported a longer median wait time than Ontario across all time periods. However, this difference has generally decreased over the study period, from a striking 51% difference (105 vs. 52 days) in the pre-pandemic period to 43% (98 vs. 56 days) in wave 1, to 47% (77 vs. 41 days) in wave 2, and to 22% (69 vs. 54 days) in wave 3, before rising again to 64% (77 vs. 28 days) in wave 4. These changes in wait times during the COVID-19 pandemic may also support our previous assertion that the strategies implemented in Alberta may have been effective in increasing surgical care access and reducing wait times.

While healthcare capacity, and associated policies may explain some of the similarities and differences between provinces, differences in patient demographic characteristics cannot be discounted. Pre-pandemic differences in healthcare utilization for surgical patients between the two provinces showed that Ontario had lower median ICU hours (40.0 vs. 54.3), hospital length of stay (2 vs. 26 days) and resource intensity for a surgical hospital admission (1.14 vs. 1.95) compared to Alberta. These pre-pandemic differences may be attributed to patient demographics since the Alberta cohort included more high-priority, cancer, and cardiovascular surgical patients who are known to be intensive users of inpatient care resources (25–28). In contrast, more than three-quarters of Ontario surgeries scheduled in pre-pandemic period were classified as low-priority. Many of these low-priority elective surgeries do not require long hospital stays, and while in hospital require far less resources than patients receiving more complex, high-priority procedure, especially cardiovascular and cancer surgeries (25–28). The contrasting differences in hospital and ICU length of stay between provinces (i.e., Alberta reported a large decrease in median hospital and ICU stays for surgical patients, while Ontario reported an increase in ICU length of stay and a stable hospital length of stay) may also be related to differences in surgical care pathways in the two provinces. For instance, Alberta implemented the Enhanced Recovery After Surgery (ERAS) Society guidelines (29) provincially in 2013 which have been shown to be effective at decreasing the hospital length of stay, internationally (30) and locally (31, 32). As of the start of COVID-19, ERAS guidelines have been introduced to nine major hospitals (29). While patient demographic characteristics and care pathways may explain some of the observed difference in healthcare resource use, additional studies are needed to assess the specific contribution of each of these on healthcare resource use during public health emergencies. Moreover, future work should explore how existing guidelines and care pathways can be adapted to improve surgical care delivery during public health emergencies and tailored to improve precision healthcare delivery during times of healthcare constraint.

While this study has strengths, including comparable methods and population-based data in both provinces, there are also limitations which should be considered. In the first-part Ontario analysis, we did not explore any moderation or mediation effects of variables. Future research can apply the appropriate techniques to provide insights (22, 33). There were unmeasured confounders such as proximity to hospital that we did not account for; beyond staying consistent with the Alberta study (6), we believe these variables to have minimal impact on the main study findings, based on prior literature (34–36) and the absence of significant differences between pre-pandemic and pandemic scheduled surgeries in the current analysis. In the second-part comparison, we were unable to isolate the true inter-provincial differences from those caused by the availability of data and study settings. For example, the definition we used for the four waves of COVID-19 in Ontario was slightly different from that of Alberta. We were also unable to map the differences to specific public health policy. Overall, the time period between decision-to-treat and operation represents a segment of a patient's full surgical journey. Future research is required to elucidate the pandemic impact on time from referral to first clinician consultation. Both provinces are single-payer healthcare systems, meaning that our results have limited generalizability to other jurisdictions. Finally, by using the surgical information databases we only captured patients who were able to see a surgeon and have a procedure booked; future study needs to provide insights for the healthcare system to find the “missing” patients.

## 5 Conclusion

This large multi-jurisdictional comparative study used deterministically linked health administrative datasets in Ontario and Alberta on adult patients with a non-urgent surgery scheduled in 2018–2021. Pronounced differences were found between provinces in the rate of scheduled surgeries, the proportion of surgeries completed (as of December 2021), the wait time for those who had their surgery completed and some measures of healthcare utilization. The observed inter-provincial differences in surgical care during the COVID-19 pandemic may be related to healthcare system capacity and patient demographics, but given differences in healthcare delivery and policies, the potential role of novel approaches to surgical care delivery (including centralized referrals and ERAS guidelines) may have been additional drivers of these differences. Future research focused on examining the effectiveness of these approaches to surgical delivery, which could be leveraged during future public health emergencies, is needed.

## Data Availability

The data analyzed in this study is subject to the following licenses/restrictions: the dataset from this study is held securely in coded form at ICES (formerly the Institute for Clinical Evaluative Sciences). While legal data sharing agreements between ICES and data providers (e.g., healthcare organizations and government) prohibit ICES from making the dataset publicly available, access may be granted to those who meet pre-specified criteria for confidential access, available at www.ices.on.ca/DAS (das@ices.on.ca). The full dataset creation plan and underlying analytic code are available from the authors upon request, understanding that the computer programs may rely upon coding templates or macros that are unique to ICES and are therefore either inaccessible or may require modification. Requests to access these datasets should be directed to antoine.eskander@mail.utoronto.ca.
