# Supplementary material for: COVID-related delays in non-urgent adult surgeries: comparing population-based results from two Canadian provinces
Source: Front Surg. 2025 Jul 28;12:1591265. doi: 10.3389/fsurg.2025.1591265 (PMC12336234; doi:10.3389/fsurg.2025.1591265)
Supplement: Supplementary file 1 [file Datasheet1.pdf]

Supplementary Material

Supplemental Table 1 Data sources

Supplemental Figure 1 Creation of the Ontario study cohort

Supplemental Table 2 Surgery completion in Ontario vs. Alberta

Supplemental Table 3 Logistic regression of surgery completion on index date period, patient age, patient sex, region, comorbidity, surgery type, and surgery priority, in Ontario

Supplemental Table 4 Impact of index date period on surgery completion, by surgery type in Ontario

Supplemental Table 5 Surgical wait times in Ontario vs. Alberta

Supplemental Table 6 Multivariable median regression of surgical waiting time on index date period, patient age, patient sex, region, comorbidity, surgery type, and surgery priority, in Ontario

Supplemental Table 1 Data sources

Database	Description
**Registered Persons Database (RPDB)**	The RPDB is an ICES database derived from all administrative data sources and provides demographic data including age, patient residence, vital status, date of last contact with the healthcare system, and OHIP eligibility. It contains the basic demographic information about anyone who has ever received an Ontario health card number.
**Wait Time Information System (WTIS)**	The WTIS is a web-based application that enables Ontario hospitals to capture wait times information and stakeholders to standardize wait time tracking related to surgery, diagnostic imaging and alternative level of care. It was developed and implemented by Ontario Health as part of the Ministry of Health and Long-Term Care's Wait Times Strategy and Emergency Room/Alternate Level of Care Information Strategy. In near real time, the WTIS captures and reports on wait times for surgical procedures, including Wait 1 (from referral to first consultation) and Wait 2 (from decision-to-treat to surgery)
**Ontario Registrar General (ORG)**	The ORG contains gold standard vital status data for all Ontarians. According to the Vital Statistics Act, it is mandatory to register all deaths occurring in the province.
**Ontario Health Insurance Plan (OHIP) Claims Database**	The OHIP claims database contains all physician billing records including information on diagnoses and services provided.
**Discharge Abstract Database (DAD)**	The DAD is a data holding of the Canadian Institute for Health Information (CIHI) that captures administrative, clinical, and demographic information on hospital discharges including deaths, sign-outs, and transfers. Every time an acute care hospital discharges a patient, the hospital submits an electronic record to CIHI that contains the patient's demographic information as well as diagnostic and treatment data.
**Same Day Surgery (SDS)**	The SDS is a CIHI database that holds the records of all day surgeries.
**National Ambulatory Care Reporting System (NACRS)**	The NACRS is a CIHI database that contains data for hospital- and community-based ambulatory care including day surgery, outpatient and community-based clinic visits (including dialysis and cancer visits), and emergency department visits. CIHI receives data directly from participating facilities, their respective regional health authorities or the Ministry of Health.

Supplemental Figure 1 Creation of the Ontario study cohort

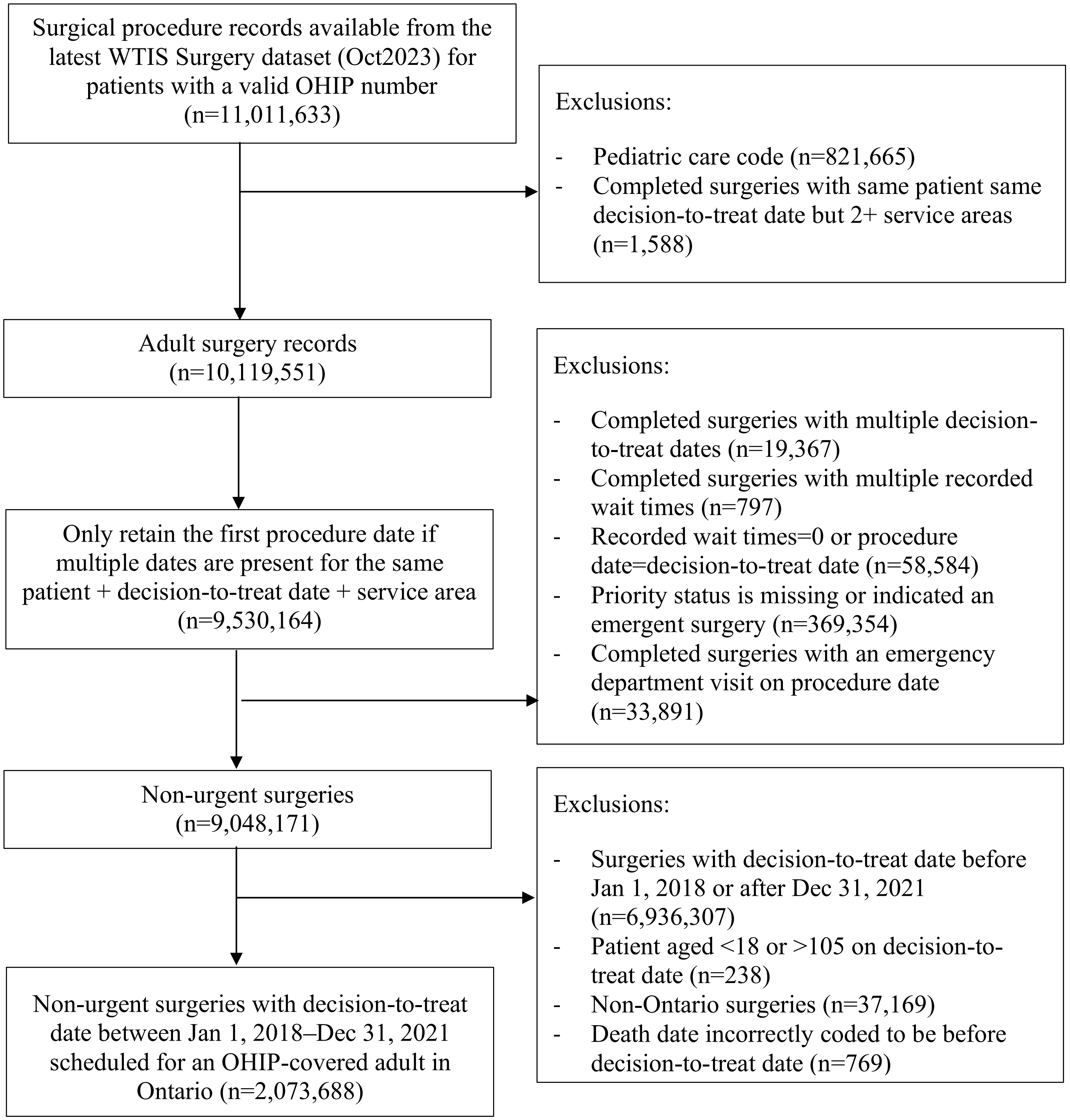

WTIS, Wait Time Information System; OHIP, Ontario Health Insurance Plan

Supplemental Table 2 Surgery completion in Ontario vs. Alberta

	Ontario		Alberta	
**Index (decision-to-treat) time period**	**Scheduled**	**Completed as of Dec 31, 2021**	**Scheduled**	**Completed as of Dec 31, 2021**
**Pre-pandemic**	1243209	1141977 (91.9%)	115537	91473 (79.2%)
**Pandemic**				
**Wave 1**	154145	137876 (89.4%)	33648	26530 (78.8%)
**Wave 2**	257798	227733 (88.3%)	52580	41668 (79.2%)
**Wave 3**	208064	164838 (79.2%)	29229	22186 (75.9%)
**Wave 4**	210472	105453 (50.1%)	28683	19204 (66.9%)
**Overall (Waves 1-4)**	830479	635900 (76.6%)	144140	109588 (76.0%)
**Total**	2073688	1777877 (85.7%)	259677	201061 (77.4%)

Decision-to-treat (index) date was used to classify each surgical procedure into the following index time periods for Alberta and Ontario: Alberta: pre-pandemic (January 1, 2018–February 29, 2020), Wave 1 (March 1, 2020–August 22, 2020), Wave 2 (August 23, 2020–March 20, 2021), Wave 3 (March 21, 2021–July 17, 2021), and Wave 4 (July 18, 2021–December 31, 2021); Ontario: pre-pandemic (January 1, 2018–February 29, 2020), Wave 1 (March 1, 2020–August 29, 2020), Wave 2 (August 30, 2020–February 27, 2021), Wave 3 (February 28, 2021–July 31, 2021), and Wave 4 (August 1, 2021–December 31, 2021). All surgeries were followed from the index date to procedure date, death date, or December 31, 2021, whichever occurred first.

Supplemental Table 3 Logistic regression of surgery completion on index date period, patient age, patient sex, region, comorbidity, surgery type, and surgery priority, in Ontario

Variables	Referent	aOR	95% CI	*P* Value
Time period of decision-to-treat				
Wave 1	Pre-pandemic	0.682	0.670 – 0.694	<0.01
Wave 2		0.641	0.632 – 0.650	<0.01
Wave 3		0.312	0.308 – 0.316	<0.01
Wave 4		0.078	0.077 – 0.079	<0.01
Age at the index, year				
50–64	18–49	1.071	1.059 – 1.084	<0.01
65–74		1.046	1.032 – 1.060	<0.01
75 and above		0.946	0.933 – 0.960	<0.01
Female	Male	1.00	0.991 – 1.010	0.93
Region of institution				
West	Toronto	0.875	0.865 – 0.886	<0.01
Central		0.881	0.870 – 0.893	<0.01
East		0.797	0.787 – 0.808	<0.01
Northeast		1.032	1.010 – 1.056	<0.01
Northwest		0.779	0.756 – 0.803	<0.01
Charlson Comorbidity Index				
1	0 or no records in past 5 years	0.970	0.955 – 0.986	<0.01
2 or above		0.922	0.910 – 0.934	<0.01
Surgery type				
Gynecologic	General surgery	0.871	0.855 – 0.886	<0.01
Neurologic		0.877	0.843 – 0.912	<0.01
Oncology		2.347	2.295 – 2.399	<0.01
Ophthalmic		1.146	1.129 – 1.164	<0.01
Oral, maxillofacial, dentistry		0.510	0.495 – 0.526	<0.01
Orthopedic		0.686	0.676 – 0.696	<0.01
Otolaryngology		0.680	0.666 – 0.694	<0.01
Plastic and reconstructive		0.925	0.904 – 0.947	<0.01
Thoracic		0.829	0.771 – 0.893	<0.01
Urologic		1.107	1.086 – 1.128	<0.01
Vascular		1.244	1.201 – 1.289	<0.01
Surgery priority level at the index				
Priority II	Priority IV	2.136	2.087 – 2.185	<0.01
Priority III		1.448	1.432 – 1.464	<0.01

aOR, adjusted odds ratio; CI, confidence interval.

Supplemental Table 4 Impact of index date period on surgery completion, by surgery type in Ontario

Surgery type	Comparison	aOR	95% CI	*P*-value
General	1 vs. Pre	0.803	0.767 – 0.841	<0.01
	2 vs. Pre	0.728	0.702 – 0.755	<0.01
	3 vs. Pre	0.392	0.379 – 0.406	<0.01
	4 vs. Pre	0.098	0.095 – 0.101	<0.01
Gynecologic	1 vs. Pre	0.653	0.620 – 0.688	<0.01
	2 vs. Pre	0.612	0.587 – 0.639	<0.01
	3 vs. Pre	0.323	0.311 – 0.336	<0.01
	4 vs. Pre	0.075	0.073 – 0.078	<0.01
Neurologic	1 vs. Pre	0.688	0.591 – 0.800	<0.01
	2 vs. Pre	0.531	0.470 – 0.600	<0.01
	3 vs. Pre	0.283	0.252 – 0.317	<0.01
	4 vs. Pre	0.088	0.080 – 0.098	<0.01
Oncology	1 vs. Pre	0.722	0.675 – 0.772	<0.01
	2 vs. Pre	0.971	0.906 – 1.041	0.41
	3 vs. Pre	0.739	0.691 – 0.790	<0.01
	4 vs. Pre	0.113	0.109 – 0.118	<0.01
Ophthalmic	1 vs. Pre	0.633	0.605 – 0.661	<0.01
	2 vs. Pre	0.493	0.479 – 0.509	<0.01
	3 vs. Pre	0.193	0.188 – 0.199	<0.01
	4 vs. Pre	0.045	0.044 – 0.046	<0.01
Oral, maxillofacial, dentistry	1 vs. Pre	0.661	0.582 – 0.751	<0.01
	2 vs. Pre	0.689	0.628 – 0.755	<0.01
	3 vs. Pre	0.406	0.371 – 0.445	<0.01
	4 vs. Pre	0.105	0.096 – 0.114	<0.01
Orthopedic	1 vs. Pre	0.696	0.672 – 0.721	<0.01
	2 vs. Pre	0.649	0.632 – 0.667	<0.01
	3 vs. Pre	0.301	0.294 – 0.309	<0.01
	4 vs. Pre	0.070	0.068 – 0.072	<0.01
Otolaryngology	1 vs. Pre	0.632	0.589 – 0.679	<0.01
	2 vs. Pre	0.592	0.561 – 0.626	<0.01
	3 vs. Pre	0.294	0.280 – 0.310	<0.01
	4 vs. Pre	0.082	0.078 – 0.086	<0.01
Plastic and reconstructive	1 vs. Pre	0.633	0.583 – 0.688	<0.01
	2 vs. Pre	0.639	0.598 – 0.682	<0.01
	3 vs. Pre	0.324	0.305 – 0.345	<0.01
	4 vs. Pre	0.097	0.092 – 0.102	<0.01
Thoracic	1 vs. Pre	0.749	0.551 – 1.017	0.06
	2 vs. Pre	0.687	0.540 – 0.875	<0.01
	3 vs. Pre	0.250	0.202 – 0.308	<0.01
	4 vs. Pre	0.109	0.091 – 0.131	<0.01
Urologic	1 vs. Pre	0.689	0.649 – 0.731	<0.01
	2 vs. Pre	0.745	0.709 – 0.784	<0.01
	3 vs. Pre	0.434	0.414 – 0.455	<0.01
	4 vs. Pre	0.134	0.129 – 0.140	<0.01
Vascular	1 vs. Pre	0.750	0.665 – 0.845	<0.01

Surgery type	Comparison	aOR	95% CI	*P*-value
	2 vs. Pre	0.826	0.742 – 0.921	<0.01
	3 vs. Pre	0.541	0.487 – 0.600	<0.01
	4 vs. Pre	0.171	0.158 – 0.186	<0.01

We repeated the multivariable logistic regression model on each surgery type to derive the surgery type-specific odds ratio of completion (as of Dec 31, 2021). The following covariates were adjusted in the regression model: age, sex, region of the institution, comorbidity, and priority level of surgery. All covariates were measured at the index date. Surgical procedures were treated as independent events. aOR, adjusted odds ratio; CI, confidence intervals.

Supplemental Table 5 Surgical wait times in Ontario vs. Alberta

**Ontario**			
**Index (decision-to-treat) time period**	**Number of scheduled surgeries completed as of Dec 31, 2021**	**Mean (SD), d**	**Median (Q1-Q3), d**
Pre-pandemic	114197	89.7 ± 108.3	52 (25 – 110)
Pandemic			
Wave 1	137876	96.1 ± 106.2	56 (20 – 139)
Wave 2	227733	75.3 ± 84.8	41 (20 – 94)
Wave 3	164838	72.0 ± 61.2	54 (21 – 109)
Wave 4	105453	34.4 ± 25.3	28 (15 – 47)
Overall (Waves 1-4)	635900	72.2 ± 80.4	41 (20 – 96)
**Alberta**			
**Index (decision-to-treat) time period**	**Number of scheduled surgeries completed as of Dec 31, 2021**	**Mean (SD), d**	**Median (Q1-Q3), d**
Pre-pandemic	91473	154.9 ± 154.5	105 (43 – 218)
Pandemic			
Wave 1	26530	138.6 ± 133.7	98 (36 – 204)
Wave 2	41668	120.9 ± 118.8	77 (34 – 173)
Wave 3	22186	111.3 ± 102.8	69 (30 – 180)
Wave 4	19204	92.5 ± 73.1	77 (30 – 139)
Overall (Waves 1-4)	109588	118.3 ± 114.0	80 (33 – 173)

Decision-to-treat date was used to classify each surgical procedure into the following index time periods for Alberta and Ontario: Alberta: pre-pandemic (January 1, 2018–February 29, 2020), Wave 1 (March 1, 2020–August 22, 2020), Wave 2 (August 23, 2020–March 20, 2021), Wave 3 (March 21, 2021–July 17, 2021), and Wave 4 (July 18, 2021–December 31, 2021); Ontario: pre-pandemic (January 1, 2018–February 29, 2020), Wave 1 (March 1, 2020–August 29, 2020), Wave 2 (August 30, 2020–February 27, 2021), Wave 3 (February 28, 2021–July 31, 2021), and Wave 4 (August 1, 2021–December 31, 2021). For both provinces, surgical completion status was observed up to December 31, 2021 or until the date of death (whichever occurred first).

SD, standard deviation; d, day; Q1, 1st quartile; Q3, third quartile.

Supplemental Table 6 Multivariable median regression of surgical waiting time on index date period, patient age, patient sex, region, comorbidity, surgery type, and surgery priority, in Ontario

Variables	Referent	Adjusted difference in median, d	95% CI	*P* Value
Time period of decision-to-treat				
Wave 1		8.79	8.38 to 9.21	<0.01
Wave 2	Pre-pandemic	-6.58	-6.79 to -6.37	<0.01
Wave 3		5.32	5.03 to 5.63	<0.01
Wave 4		-15.96	-16.20 to -15.72	<0.01
Age at the index, year				
50–64	18–49	2.92	2.70 to 3.14	<0.01
65–74		5.40	5.17 to 5.64	<0.01
75 and above		4.03	3.78 to 4.28	<0.01
Female	Male	1.23	1.06 to 1.39	0.01
Region of institution				
West		8.80	8.61 to 9.00	<0.01
Central		3.37	3.16 to 3.58	<0.01
East	Toronto	9.64	9.41 to 9.87	<0.01
Northeast		4.40	4.05 to 4.74	<0.01
Northwest		7.98	7.35 to 8.60	<0.01
Charlson Comorbidity Index	0 or no records in past 5 years			
1		-0.85	-1.16 to -0.53	<0.01
2 or above		-2.43	-2.59 to -2.26	<0.01
Surgery type				
Gynecologic		11.11	10.75 to 11.48	<0.01
Neurologic		4.31	3.64 to 4.97	<0.01
Oncology		-11.86	-12.08 to -11.63	<0.01
Ophthalmic		18.34	17.92 to 18.75	<0.01
Oral, maxillofacial, dentistry	General surgery	14.71	13.84 to 15.58	<0.01
Orthopedic		29.40	29.01 to 29.80	<0.01
Otolaryngology		20.34	19.76 to 20.93	<0.01
Plastic and reconstructive		2.19	1.75 to 2.63	<0.01
Thoracic		-5.66	-6.49 to -4.92	<0.01
Urologic		-7.58	-7.86 to -7.30	<0.01
Vascular		-8.28	-8.66 to -7.91	<0.01
Surgery priority level at the index				
Priority II	Priority IV	-18.40	-18.56 to -18.25	<0.01
Priority III		-37.34	-37.56 to -37.11	<0.01

CI, confidence interval; d, day.
